# FISH analysis of 107 prostate cancers shows that *PTEN* genomic deletion is associated with poor clinical outcome

**DOI:** 10.1038/sj.bjc.6603924

**Published:** 2007-08-14

**Authors:** M Yoshimoto, I W Cunha, R A Coudry, F P Fonseca, C H Torres, F A Soares, J A Squire

**Affiliations:** 1 Division of Applied Molecular Oncology, Ontario Cancer Institute, Princess Margaret Hospital, Toronto, Ontario, M5G 2M9, Canada; 2 Departamento de Patologia, Centro de Tratamento e Pesquisa, Hospital do Câncer, A.C. Camargo, São Paulo, 01509 010, Brazil; 3 Serviço de Urologia, Departamento de Cirurgia Pélvica, Hospital do Câncer, A.C. Camargo, São Paulo, 01509 010, Brazil; 4 Instituto de Matemática e Estatística, Universidade de São Paulo, São Paulo, 01509 010, Brazil; 5 Department of Medical Biophysics, Faculty of Medicine, University of Toronto, M5G 2M9, Canada

**Keywords:** interphase FISH, *PTEN* haploinsufficiency, prognostic biomarker, PSA, biochemical recurrence

## Abstract

This study examines the clinical impact of *PTEN* genomic deletions using fluorescence *in situ* hybridisation (FISH) analysis of 107 prostate cancers, with follow-up information covering a period of up to 10 years. Tissue microarray analysis using interphase FISH indicated that hemizygous *PTEN* losses were present in 42/107 (39%) of prostatic adenocarcinomas, with a homozygous *PTEN* deletion observed in 5/107 (5%) tumours. FISH analysis using closely linked probes centromeric and telomeric to the *PTEN* indicated that subband microdeletions accounted for ∼70% genomic losses. Kaplan–Meier survival analysis of *PTEN* genomic losses (hemizygous and homozygous deletion *vs* not deleted) identified subgroups with different prognosis based on their time to biochemical relapse after surgery, and demonstrated significant association between *PTEN* deletion and an earlier onset of disease recurrence (as determined by prostate-specific antigen levels). Homozygous *PTEN* deletion was associated with a much earlier onset of biochemical recurrence (*P*=0.002). Furthermore, *PTEN* loss at the time of prostatectomy correlated with clinical parameters of more advanced disease, such as extraprostatic extension and seminal vesicle invasion. Collectively, our data indicates that haploinsufficiency or *PTEN* genomic loss is an indicator of more advanced disease at surgery, and is predictive of a shorter time to biochemical recurrence of disease.

Prostate cancer is the most commonly diagnosed malignancy in men in the North America and the third leading cause of cancer-related mortality after lung and colorectal cancer in males aged 40 years and older (Jemal *et al*, 2006). In spite of significant progress in its clinical management, comparatively little is known about the disease aetiology. Widely used biochemical, histopathological and clinical criteria, for example prostate-specific antigen (PSA) level, Gleason score, and the clinical tumour stage, have demonstrated a significant variability in predicting subgroups of prostate cancer patients with distinct clinical outcome (Miller *et al*, 2001; DeMarzo *et al*, 2003; Glinsky *et al*, 2004). As such, there is an absolute necessity to improve the current patient stratification methods using biomarkers identified through studies of prostate cancer genomics.

Previous cytogenetic and genomic profiling analyses have identified several tumour-associated chromosomal rearrangements in the initial stages of sporadic primary prostate cancer, consisting predominantly of losses (Visakorpi *et al*, 1995; Verhagen *et al*, 2000; Elo and Visakorpi, 2001). For example, well-recognised changes in early prostatic carcinogenesis include loss of 8p, 6q, 10q, 13q, 16q and 18q, and gain of 8q (Qian *et al*, 1998; Zitzelsberger *et al*, 2001; Kasahara *et al*, 2002; Wolf *et al*, 2004; van Dekken *et al*, 2004; Hughes *et al*, 2006; Ribeiro *et al*, 2006a, 2006b). The importance of genomic rearrangements in prostate cancer was emphasised by the discovery of recurrent translocations in 40–60% of prostate carcinoma and in 21% of the presumed premalignant lesion, high-grade prostatic intraepithelial neoplasia (HPIN), involving the *TMPRSS2* gene with members of the erythroblast transformation-specific (*ETS*) transcription factor family (Tomlins *et al*, 2005, 2006; Ahlers and Figg, 2006; Cerveira *et al*, 2006; Perner *et al*, 2006; Soller *et al*, 2006; Wang *et al*, 2006; Yoshimoto *et al*, 2006b). Indeed, HPIN lesions reveal similar genetic features to those found in prostate carcinomas, including the loss of 8p, gain of 8q (Hughes *et al*, 2006; Ribeiro *et al*, 2006b), and genomic losses of chromosome 10 (Hughes *et al*, 2006; Ribeiro *et al*, 2006b). More detailed investigation of the 10q losses using fluorescence *in situ* hybridisation (FISH) and tissue microarrays (TMAs) showed that *PTEN* microdeletions were present in 68% of carcinomas and in 23% of HPIN lesions found in radical prostatectomies. The detection of *PTEN* deletion in HPIN suggested that somatic haploinsufficiency *per se* might be an early pivotal step in the transition from HPIN to invasive carcinoma (Yoshimoto *et al*, 2006a).

PTEN plays an important role in the modulation of the phosphatidylinositol-3-kinase (PI3K) pathway by catalysing degradation of phosphatidylinositol-(3,4,5)-trisphosphate generated by PI3K (Besson *et al*, 1999). Phosphatidylinositol-(3,4,5)-trisphosphate activates the protein kinase Akt and its regulator PDK1 (Goberdhan and Wilson, 2003), which then modulates a number of downstream targets with important roles in apoptosis and the cell-cycle progression, including BAD (Datta *et al*, 1997), CASP3 and CASP9 (Cardone *et al*, 1998), MDM2 (Ashcroft *et al*, 2002), mTOR (Majumder *et al*, 2004), the forkhead family of transcription factors (FKHR) (Brunet *et al*, 1999) and p27 (Graff *et al*, 2000). The lack of inhibition of these pathways by *PTEN* inactivation is associated with high Gleason score (Koksal *et al*, 2004) and tumour progression in prostate cancer (Koksal *et al*, 2004; Bertram *et al*, 2006).

The occurrence of *PTEN* mutation in prostate cancer is considered common, with reported frequencies (based on relatively small sample sizes), ranging from 30 to 60% (Whang *et al*, 1998; Kwabi-Addo *et al*, 2001). Given the high frequency of PTEN inactivation by genomic deletion in prostate cancer using FISH methods (Yoshimoto *et al*, 2006a), it has become more practical to conduct much larger correlative studies of patient outcome in situations when the *PTEN* gene is either lost, or retained in tumours. Such findings would further implicate a role for *PTEN* haploinsufficiency in poor prognosis and tumour progression. Moreover, knowledge of the *PTEN* deletion in the primary tumour, in addition to current clinico-pathological features, might be of value when selecting the optimum treatment for a particular patient.

## MATERIALS AND METHODS

### Tissue specimens

The collection of tissue specimens, clinical and follow-up data was obtained and handled in accordance with the Hospital do Câncer Research Ethics guidelines (São Paulo, Brazil). Archival formalin-fixed, paraffin-embedded tissues were obtained from 107 radical prostatectomies performed between 1997 and 2000 at the Hospital do Câncer, AC Camargo, São Paulo, Brazil. For control purposes, 10 non-neoplastic prostate tissue samples were obtained from patients undergoing surgery solely for benign prostate hyperplasia. The prostate cancer cohort comprising 107 tumour samples and control specimens were sampled using a 0.6 mm diameter tissue core distributed on TMA slide. Adjacent haematoxylin and eosin (H&E)-stained section was reviewed by two pathologists to determine the presence and extent of morphologically representative areas of the original tumours in each tissue core and Gleason grading. The clinico-pathological (TNM) stage and Gleason scores (range from 4 to 9) for each case were obtained from the medical and surgical pathology reports. The size of tumour was based on assessment of total surface area of the gland examined histologically involved by carcinoma. Preoperative PSA level was available for all patients and the PSA non-failure was defined as PSA remaining below 0.2 ng ml^−1^ after radical prostatectomy. Recurrence-free interval was defined as the time between date of surgery and the date of first PSA increase above 0.2 ng ml^−1^.

A separate evaluation of *PTEN* deletion status was also performed using a study group of clinically distinct prostate cancers in which inclusion criteria were the availability of paraffin-embedded formalin-fixed tissue from both an initial primary adenocarcinoma surgical specimen and from metastatic prostate adenocarcinoma in the regional lymph node metastases (Hospital do Câncer, AC Camargo, São Paulo, Brazil). A cohort of 10 such paired tumour samples were identified. Adjacent H&E-stained section was reviewed by two pathologists to determine the presence and extent of morphologically representative areas of the original tumours in each tissue. Blood preoperative PSA levels ranged from 10 to 84 ng ml^−1^ within this cohort.

### Follow-up studies

The 107 patients in the study group were followed up for a period of up to 10 years subsequent to the initial surgery. The median age at diagnosis was 63 years (range from 41 to 76). At the end of the follow-up period for each patient, 58 (54%) had a biochemical recurrence within the period of 0.79–86.28 months after initial surgery. Metastatic progression occurred in 10 patients at a median follow-up of 36.21 months from initial surgery. Survival time for the remaining 49 disease-free cases varied from 13.85 to 127.60 months. The median overall survival for the entire cohort of patients was 80.65 months. All relevant clinico-pathological features of the 107-tumour cohort are summarised in Table 1.

### FISH

Dual-colour FISH on paraffin-embedded tumour tissue was performed using commercially available DNA probes for cytoband 10q23 (Spectrum Orange *PTEN* locus-specific probe) and region 10p11.1-q11.1 (Spectrum Green centromere of chromosome 10 probe) (LSI *PTEN*/CEP 10 – Vysis Inc., Downers Grove, IL, USA). The *PTEN* genomic probe spans 368 kb and starts 166 kb from 5′ end of the gene and extends 98 kb past the 3′ end of the gene. Histologic tissue sections of 5 *μ*m were deparaffinised with a series of xylene before immersion in 100% ethanol. The slide was placed in a 2 × SSC solution at 75°C for 20 min, following by treatment in 0.25 mg ml^−1^ Proteinase K (Roche Diagnostics, Indianapolis, IN, USA) at 45°C for 20 min. The sections were dehydrated in a graded series of ethanol, and washed in 2 × SSC. Dual-colour probes and target DNA were co-denatured at 80°C for 10 min. Post-hybridisation procedures were performed by 1.5 M urea/0.1 × SSC solution at 45°C for 30 min, 2 × SSC and a graded ethanol series.

Sequential tri-colour FISH method was applied to the prostate cancer TMA to map the flanking regions of genomic loss associated with *PTEN* deletions in tumours. The following bacterial artificial chromosome (BAC) clones were used. BACs located at: (a) 10q23.2 (88.2–88.7 Mb, 900 kb upstream from the 5′ *PTEN* gene locus): RP11-141D8, RP11-52G13 and RP11-420K10; (b) 10q23.2 (89.4–89.5 Mb, 100 kb on the centromeric side of the 5′ *PTEN*): RP11-11O21; (c) 10q23.31 (89.6–89.7 Mb, *PTEN* gene locus): RP11-383D9; (d) 10q23.33 (96.2–96.6 Mb, 6.6 Mb on the telomeric side of the 3′ *PTEN* gene locus): RP11-119K6, RP11-90J1 and RP11-466J14; (e) 10q25.1 (108.9–109.3 Mb, 19.3 Mb downstream from the 3′ *PTEN* gene locus): RP11-246B13, RP11-49H17 and RP11-432B10. The position and name of the BAC clones were taken from the Human March 2006 assembly of the UCSC Genome Browser 1. DNA was extracted and labelled with either Spectrum Green-dUTP, SpectrumOrange-dUTP (Vysis) or DEAC-dUTP (PerkinElmer Life and Analytical Sciences, Boston, MA, USA) using the Vysis nick-translation kit (Vysis). Labelling of probes was done as described previously (Merscher *et al*, 1997; Sirvent *et al*, 2000). The chromosome localisation of all BAC clones was confirmed by both normal metaphase and tri-colour FISH analysis.

### Data analysis

*PTEN* copy number was evaluated for each probe by counting spots in a range from 50 to 100 non-overlapped, intact interphase nuclei per tumour tissue core. 4′,6-Diamidino-2-phenylindole, dihydrochloride staining of nuclei with reference to the corresponding H&E-stained tissue identified the areas of adenocarcinoma. Based on hybridisation in 10 control cores (data not shown), hemizygous deletion of *PTEN* were defined as >20% (mean+3 s.d. in non-neoplastic controls) of tumour nuclei containing one *PTEN* locus signal and by the presence of CEP 10 signals. Homozygous deletion of *PTEN* was exhibited by the simultaneous lack of the both *PTEN* locus signals and by the presence of control signals (Mezzelani *et al*, 1999; Kawai *et al*, 2004; Korshunov *et al*, 2005; Ventura *et al*, 2006) in >30% of cells (Korshunov *et al*, 2005).

### Statistical analysis of *PTEN* deletion in 107-prostate cancer TMA

Fluorescence *in situ* hybridisation findings for *PTEN* deletions were correlated in a univariate and multivariate approaches with clinical and pathologic features of disease aggressiveness. Initially, presence and absence of deletion by FISH was correlated with determinants of disease mortality and morbidity including PSA, Gleason score and extraprostatic extension, as well as clinically relevant end points such as time to biochemical relapse, and the development time of metastases following the definitive treatment. For prediction of 5-year biochemical risk failure, *PTEN* status (not deleted, hemi- or homozygous deletion) was compared with all relevant clinico-pathological features (Table 1). Univariate and multivariate analyses of biochemical risk failure were studied by Cox Proportional Hazard model (Tables 2 and 3, respectively). A significant correlation between two parameters was taken at the 95% confidence interval. *P*-values <0.05 were considered significant. In addition, the survival rate was estimated by applying the Kaplan–Meier method. The end point for calculating the survival time was defined by the time from radical prostatectomy until the occurrence of metastasis or PSA determined by the biochemical recurrence. Correlation of *PTEN* copy number changes with Gleason score was tested using Pearson's *χ*^2^ statistic. All calculations were performed using Stata 9.1.

## RESULTS

### *PTEN* deletion analysis in 107-prostate cancer TMA

**Figure 1:**
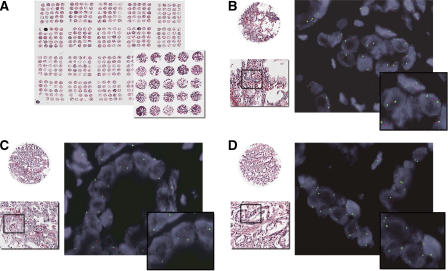
*PTEN* probe enumeration and representative dual-colour FISH images are shown for prostate cancer TMA. (**A**) Digital images of H&E-stained tissue microarray from 107 prostate cancer samples show areas of prostatic adenocarcinoma at low magnification and at × 20 magnification (rectangle) (ScanScope® System, Aperio Technologies, Inc., Vista, CA, USA). (**B**–**D**) A magnified H&E area is displayed in a tissue core with accompanying FISH for the *PTEN* locus. The rectangles show the main FISH image high magnification. (**B**) The FISH image shows two signals of both red signals (10q23/*PTEN* locus) and green signals (CEP 10) in most of the nuclei indicating no deletion of *PTEN* in tumour cells. (**C**) The FISH image shows tumour cells with single red signal for 10q23/*PTEN* locus in most of the nuclei and paired green signals for CEP 10 indicating hemizygous deletion of 10q23/*PTEN* locus in prostate cancer. (**D**) Representative FISH image of homozygous deletion in prostate cancer shows absence of red signal for 10q23/*PTEN* locus in most of the nuclei and retained green signals for CEP 10.

To investigate whether loss of *PTEN* as determined by interphase FISH indicated a greater prevalence in poor prognosis patients, the frequency of *PTEN* deletion was investigated in the cohort of 107 tumour samples using a TMA in which anonymous annotation codes allowed interrogation of clinical outcome parameters. Hemizygous *PTEN* deletion was found in 42 of the 107 (39%) adenocarcinomas samples. As shown in Table 4, homozygous *PTEN* deletion was found in 5 of the 107 (5%) prostate adenocarcinomas. Representative images of undeleted, hemizygous and homozygous deletions are shown in Figure 1. Comprehensive FISH raw data sets for all samples is summarised in Supplementary Table.

Among the hemizygous *PTEN*-deleted tumours detected from the cohort of 107 adenocarcinomas, there were 42 patients with hemizygous *PTEN* deletion that were classified as Gleason score 4–6 (20 tumours), 7 (16 tumours) and 8–9 (6 tumours). A median tumour volume of >20% was found in 17 of the 42 adenocarcinomas. Biochemical recurrence based on PSA level was present in 28 of 42 samples. A median tumour volume of >20% was found in all five adenocarcinomas with homozygous *PTEN* deletion. In addition, early biochemical recurrence was detected in all five of these samples. A comprehensive description of the clinical parameters associated with the adenocarcinomas having hemizygous or homozygous *PTEN* deletion is summarised in Table 1.

### Statistical analysis

**Figure 2:**
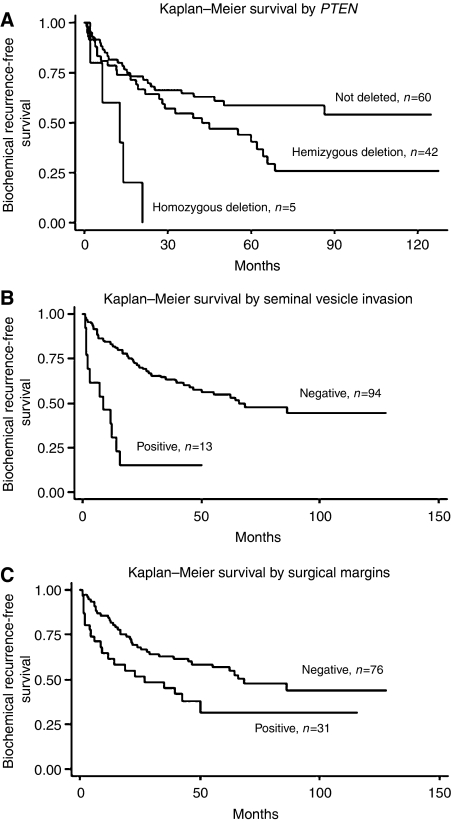
Kaplan–Meier curves illustrating biochemical recurrence-free survival among 107 prostate cancer patients defined by the status of selected clinico-pathological parameters and *PTEN* copy number changes. (**A**) PSA recurrence-free survival curve stratified by the *PTEN* locus copy number changes on prostate cancer patients. (**B**) PSA recurrence-free survival analysis stratified by seminal vesicle invasion on prostate cancer patients. (**C**) PSA recurrence-free survival analysis stratified by surgical margins on prostate cancer patients.

After acquisition of FISH data, the 107 cases were reviewed to search for potential associations between genomic loss of *PTEN*, clinical variables of disease progression and tumour histology. Univariate analysis of biochemical risk failure was significant for perineural invasion, extraprostatic extension, seminal vesicle invasion, Gleason score, preoperative PSA, lymph nodal invasion and *PTEN* deletion (Table 2). For prediction of 5-year biochemical risk failure by χ^2^ analysis, *PTEN* status (not deleted, hemi- or homozygous deletion) was significantly associated with disease recurrence based on PSA levels (Table 1). Other clinical parameters of aggressive disease such as extraprostatic extension, seminal vesicle and perineural invasion were also significantly associated with biochemical recurrence. By multivariate analysis (Table 3), *PTEN* deletion, extraprostatic extension and seminal vesicle invasion were observed at an independent level to explain biochemical failure. For comparison purpose, Kaplan–Meier survival analysis applying established clinical markers, such as the preoperative PSA, seminal vesicle invasion and surgical margins status, was considered to identify subgroups with different prognosis with respect to time of relapse after surgery. The estimated disease-free survival curves demonstrated significant association between *PTEN* deletion and short time based on PSA recurrence intervals. Significantly homozygous *PTEN* deletion in five tumours was associated with a much earlier onset of biochemical recurrence based on PSA values (Figure 2).

### Analysis of *PTEN* deletion in cohort of 10-paired primary adenocarcinomas and metastatic prostate adenocarcinoma in the regional lymph nodes

*PTEN* deletion frequency was determined by dual-colour FISH using paired primary prostate adenocarcinomas and metastatic adenocarcinoma in the regional lymph nodes derived from 10 patients. Overall, the presence of *PTEN* deletion was found at high frequency (9 of 10) in both paired primary and metastatic lymph nodal prostate adenocarcinoma samples in this study group. Only one patient of the 10 retained both copies of the *PTEN* locus in his primary tumour and in his metastatic lymph nodal biopsy. Hemizygous *PTEN* deletion was found in both the primary and the metastatic nodal tumour samples in four of 10 patients. Homozygous *PTEN* deletion was found in both the primary tumour and their metastatic lymph nodes in three of the 10 patients. Significantly, two of the 10 patients with a hemizygous *PTEN* deletion in their primary adenocarcinomas, had positive lymph node biopsies that had acquired a homozygous *PTEN* deletion. These findings suggest that loss of the remaining *PTEN* locus may be associated with metastasis.

### Mapping the adjacent genomic regions deleted when *PTEN* is lost

Tri-colour FISH using the BAC probes spanning the band 10q23.2, 5′ flanking *PTEN* probe on the centromeric side of the locus, *PTEN* locus (10pq23.31), and more telomeric probes mapping to 10q23.33 and 10q25.1, was performed on 10 of the 107 adenocarcinomas with hemizygous *PTEN* deletion and three of the 107 adenocarcinomas with homozygous *PTEN* deletion (Table 5). Sequential tri-colour FISH analyses from 7 of the 10 *PTEN* hemizygously deleted adenocarcinomas revealed BAC probes either side of *PTEN* were retained as two copies, indicating that a hemizygous *PTEN* deletion was usually accompanied by an interstitial microdeletion within band 10q23.2–q23.31. Among the three homozygous *PTEN* deletions, sequential tri-colour FISH often revealed the hemizygous loss of the 10q23.2, 10q23.33 and 10q25.1 signals, and homozygous loss of the 5′ flanking *PTEN* genomic region and the *PTEN* locus signals.

## DISCUSSION

The current challenge faced by prostate cancer researchers is to discover the critical genes and cognate molecular pathways responsible for the onset of neoplasia and disease progression, as well as the development of novel therapeutic strategies based on these discoveries. Initial progress in understanding the genetics of this disease started with cytogenetic and genomic analyses of primary prostate cancer tumours. Chromosomal losses of 10q suggested that *PTEN* at cytoband 10q23.3 might be a tumour suppressor gene involved in the development of prostate cancer (Whang *et al*, 1998). More recently, a single-nucleotide polymorphism mapping array in prostate cancer (Liu *et al*, 2006) has implicated the *PTEN* region to be the most frequently deleted in prostate cancer. The reported frequency of *PTEN* deletion in prostate cancer varies widely, most likely as a result of differences in tissue preparation, stage of disease, and the methodology used to detect molecular aberrations (Yoshimoto *et al*, 2006a). The heterogeneous nature of these studies has potentially obscured the clinical impact of *PTEN* loss in human prostate cancer. Our findings using interphase FISH analysis of prostatic adenocarcinoma TMAs have shown that *PTEN* deletion is an important event in tumour progression of prostate cancer. The value of analysing *PTEN* genomic losses by FISH methodologies is illustrated by its ability to distinguish both deletion events associated with homozygous *PTEN* losses in tumours. Moreover, our FISH analysis is able to predict that 70% of hemizygous *PTEN* deletion will involve an interstitial microdeletion within band 10q23.2–23.31 since flanking BAC probes were usually not deleted.

PTEN is a phosphoinositide 3-phosphatase which negatively regulates the PI3K/AKT signalling pathway (Ohigashi *et al*, 2005). Investigations to understand the role of *PTEN* loss have utilised a well-characterised animal model of human prostate cancer (Kwabi-Addo *et al*, 2001). Analysis of tumour progression in Pten (+/–) heterozygous mice, coupled with analysis of the *PTEN* gene and protein in the resulting tumours, has shown that haploinsufficiency of the *PTEN* gene promotes instability and the progression of prostate cancer (reviewed in Baker, 2007). Decreased PTEN activity has also been identified in several human cancers including prostate cancer (McMenamin *et al*, 1999; Koksal *et al*, 2004). A recent FISH and immunohistochemical PTEN analysis reported by our group showed deletions of *PTEN* at a very high frequency prostate cancer (Yoshimoto *et al*, 2006a). Other studies have demonstrated an association between decreased PTEN protein expression and a higher Gleason grade and advanced tumour stage (McMenamin *et al*, 1999; Koksal *et al*, 2004; Schmitz *et al*, 2007). Recently, complete loss of PTEN expression was observed in 26 of 112 (23%) of prostate cancer patients at the time of first diagnosis (Schmitz *et al*, 2007). Moreover, it was reported that 25 of 42 (59%) of both the neoplastic prostate glands and the invasive prostatic adenocarcinomas cells in the lymph node showed complete lack of PTEN expression, and of these 52% exhibited already loss of PTEN expression at first diagnosis. To determine whether *PTEN* genomic losses were also more prevalent in metastatic disease, as predicted by the above reports, we selected a small cohort of paired primary prostate adenocarcinomas and metastatic adenocarcinoma in the regional lymph nodes derived from the same patient. A very high frequency (90%) of *PTEN* deletion was evident in this study group, consistent with notion that loss of PTEN function is generally associated with more aggressive disease. Interestingly, two patients with hemizygous deletions in their primary tumours had homozygous deletions in their matched nodal metastatic sample. These data are in keeping with the idea that complete loss of PTEN function is associated with transition to more progressive disease (Verhagen *et al*, 2006; Schmitz *et al*, 2007).

In this study, we also demonstrate that both hemi- and homozygous *PTEN* loss is a highly significant prognostic marker for poor clinical outcome in prostate cancer. Prediction of 5-year biochemical risk failure analysis showed that *PTEN* status (not deleted, hemi- or homozygous deletion) correlated specifically with biochemical recurrence based on PSA levels. Our findings show a strong association between *PTEN* deletion and a shorter time interval to PSA recurrence. Significantly, homozygous *PTEN* deletion in five tumours was associated with a much earlier onset of biochemical recurrence based on PSA values. These tumours also had a median tumour volume typically >20%. Similar to the findings of (Schmitz *et al*, 2007) *PTEN* loss was not associated with Gleason score (Pearson's *χ*^2^ test, *P*=0.142). However, the absence of a statistical relationship in our study may have arisen because of the relatively small distribution of different Gleason scores in our cohort.

These novel findings implicate further an early role for *PTEN* haploinsufficiency and poor clinical outcome in prostate cancer. Our studies have demonstrated that the presence of *PTEN* genomic losses are frequent at diagnosis and are a significant prognostic marker for the subsequent development of clinically advanced disease. Previously, PTEN inactivation has been primarily been detected in locally advanced (Whang *et al*, 1998) or metastatic prostate cancer (Schmitz *et al*, 2007). Our new findings suggest that the acquisition of the deletion and concomitant loss of PTEN functional activity at an earlier phase in prostatic oncogenesis is an important determinant of the molecular pathways that govern a more aggressive tumour phenotype. Knowledge of the pathways downstream to PTEN and the genomic status of the *PTEN* gene in tumours must be considered in the design of future therapeutic trials of prostate cancer.

## Figures and Tables

**Table 1:** Clinico-pathological parameters from 107 prostatic adenocarcinoma patients

		***PTEN***			
**Clinico-pathological parameters**	**Number of cases**	**Not deleted**	**Hemizygous**	**Homozygous**	
Age median (min–max)	61.9 years (48–69)				
Preoperative PSA (ng ml^−1^)^a^					*P*=0.556
0.9–4.0	7 (6.7%)	3 (42.9%)	4 (57.1%)	0 (0%)	
4.1–10.0	55 (52.3%)	32 (58.2%)	22 (40%)	1 (1.8%)	
10.1–20.0	28 (26.7%)	19 (67.8%)	8 (28.6%)	1 (3.6%)	
20.1–84.0	15 (14.3%)	6 (40%)	8 (53.3%)	1 (6.7%)	
					
Median tumour volume (%)^a^					*P*=0.158
0–10.0	32 (34%)	19 (59.4)%	13 (40.6%)	0 (0%)	
10.1–20.0	19 (20.2%)	12 (63.2%)	7 (36.8%)	0 (0%)	
20.1–85.0	43 (45.8%)	21 (48.9%)	17 (39.5%)	5 (11.6%)	
					
Gleason score					*P*=0.142
Gleason 4–6	61 (57%)	39 (63.9%)	20 (32.8%)	2 (3.3%)	
Gleason 7	33 (30.8%)	16 (48.48%)	16 (48.48%)	1 (3.03%)	
Gleason 8–9	13 (12.2%)	5 (38.46%)	6 (46.15%)	2 (15.38%)	
					
Pathologic stage					*P*=0.569
pT2a	6 (5.6%)	1 (16.7%)	5 (83.3%)	0 (0%)	
pT2b	49 (45.8%)	30 (61.2%)	16 (32.7%)	3 (6.1%)	
pT3a	32 (29.9%)	18 (56.2%)	13 (40.6%)	1 (3.1%)	
pT3b	13 (12.2%)	4 (57.1%)	3 (42.9%)	0 (0%)	
pT4	7 (6.5%)	4 (57.1%)	3 (42.9%)	0 (0%)	
					
Seminal vesicle invasion					*P*=0.859
Negative	94 (87.9%)	53 (56.4%)	37 (39.4%)	4 (4.2%)	
Positive	13 (12.1%)	7 (53.8%)	5 (38.5%)	1 (7.7%)	
					
Perineural infiltration					*P*=0.517
Negative	15 (14.0%)	10 (66.7%)	5 (33.3%)	0 (0%)	
Positive	92 (86%)	50 (54.4%)	37 (40.2%)	5 (4.4%)	
					
Angiolymphatic invasion^a^					*P*=0.346
Negative	78 (77.2%)	46 (59%)	29 (37.2%)	3 (3.8%)	
Positive	23 (22.8%)	10 (43.5%)	11 (47.8%)	2 (8.7%)	
					
Capsular invasion					*P*=0.729
Negative	46 (43%)	25 (54.4%)	18 (39.1%)	3 (6.5%)	
Positive	61 (57%)	35 (57.4%)	24 (39.3%)	2 (3.3%)	
					
Extraprostatic extension^a^					*P*=0.713
Negative	83 (81.4%)	50 (60.2%)	30 (36.2%)	3 (3.6%)	
Positive	19 (18.6%)	9 (47.4%)	8 (42.1%)	2 (10.5%)	
					
Lymphonodal invasion^a^					*P*=0.812
Negative	94 (95.9%)	54 (57.4%)	35 (37.2%)	5 (5.3%)	
Positive	4 (4.1%)	2 (50%)	2 (50%)	0 (0%)	
					
Biochemical recurrence					*P*=0.005
Negative	49 (46%)	35 (71.4%)	14 (28.6%)	0 (0%)	
Positive	58 (54%)	25 (43.1%)	28 (48.3%)	5 (8.6%)	

a Values not available for all 107 cases.

Abbreviation: PSA, prostate-specific antigen.

Median overall survival was 80.65 months.

*P*-value=*χ*^2^ analysis.

**Table 2:** Univariate Cox proportional hazard analysis of biochemical failure risks (each variable predictor analysed separately)

		**BRFS**			
**Variables**	**Category**	**(5 years)**	***P*-value**	**HR**	**95% CI**
Perineural invasion	Negative	86.67	0.0038	1	Reference
	Positive	42.77		6.22	1.51–25.60
					
Extraprostatic	Negative	56.71	0.0001	1	Reference
extension	Positive	27.78		3.2	1.72–5.94
					
Seminal vesicle	Negative	54.23	0.0001	1	Reference
invasion	Positive	21.43		3.41	1.74–6.69
					
Gleason score	2–6	65.57	<0.0001	1	Reference
	7	25.74		3.04	1.72–5.37
	8–9	30.77		3.4	1.60–7.22
					
Preoperative PSA	0.9–4.0	67.69	<0.0001	1	Reference
	4.1–10	58.36		1.98	0.47–8.41
	10.1–20	51.74		2.38	0.54–10.42
	20.1–84	6.67		10.27	2.30–45.87
					
Lymphonodal	Negative	49.37	0.0068	1	Reference
invasion	Positive	25.00		4.43	1.36–14.43
					
*PTEN* deletion	Negative	58.68	0.0003	1	Reference
	Hemizygous	40.3		1.78	1.03–3.05
	Homozygous	0		6.15	2.26–16.73

Abbreviations: BRFS, biochemical recurrence free survival; CI, confidence interval; HR. hazard ratio; PSA, prostate-specific antigen.

**Table 3:** Multivariate model to biochemical failure risks by Cox logistic regression analysis

**Variables**	**Category**	**HR**	***P*-value**	**95% CI**
Extracapsular extension	Negative	1	0.004	Reference
	Positive	2.81		1.38–5.74
				
Seminal vesicle invasion	Negative	1	0.019	Reference
	Positive	2.8		1.18–6.65
				
*PTEN* deletion	Negative	1		Reference
	Hemizygous	1.96	0.022	1.10–3.49
	Homozygous	11.26	0.002	2.44–51.93

Abbreviations: CI, confidence interval; HR, hazard ratio.

Likelihood-ratio test *P*=0.0078.

**Table 4:** Analysis of *PTEN* gene copy number by FISH

***PTEN* locus**	**Number of cases (%)**
Hemizygous deletion^a^	42 (39%)
Homozygous deletion^b^	5 (5%)
Not deleted	60 (56%)

Abbreviation: FISH, fluorescence *in situ* hybridisation.

a Hemizygous deletion of *PTEN* was defined as >20% of tumour nuclei containing one *PTEN* locus signal (mean + 3 s.d. in non-neoplastic controls) (Korshunov *et al*, 2005).

b Homozygous deletions of *PTEN* was identified by the simultaneous lack of both of the *PTEN* locus signals and by the presence of CEP 10 signals in >30% of cells (Korshunov *et al*, 2005).

**Table 5:** Mapping the adjacent genomic regions deleted when *PTEN* is lost using BAC DNA probes spanning the predicted deletion interval on chromosome 10q

**TMA**	**Centromeric to *PTEN*^a^**	**5′ flanking *PTEN*^b^**	***PTEN* c**	**Telomeric to *PTEN*^d^**	**Telomeric to *PTEN*^e^**	**Predicted deletion**
19	+/+	+/−	+/−	+/+	+/+	Microdeletion
29	+/−	+/−	+/−	+/+	+/+	Small
35	+/−	+/−	+/−	+/+	+/+	Small
38	+/+	+/−	+/−	+/+	+/+	Microdeletion
39	+/+	+/−	+/−	+/+	+/+	Microdeletion
41	+/+	+/−	+/−	+/+	+/+	Microdeletion
46	+/+	+/−	+/−	+/+	+/+	Microdeletion
49	+/+	+/−	+/−	+/+	+/+	Microdeletion
91	+/+	+/−	+/−	+/−	+/+	Large
184	+/+	+/−	+/−	+/+	+/+	Microdeletion
28	+/−	−/−	−/−	+/−	+/−	Microdeletion^f^ large^g^
72	+/+	−/−	−/−	+/+	+/+	Microdeletion
88	+/−	−/−	−/−	+/−	+/−	Microdeletion^f^ Large^g^

Abbreviations: BAC, bacterial artificial chromosome; FISH, fluorescence *in situ* hybridisation.

The summarised FISH analysis described the findings from the largest clonal cell population.

+/+ no copy change; +/− hemizygous deletion; −/− homozygous deletion.

Estimated microdeletion: ∼200 kb.

Estimated small deletion: ∼7–8 Mb.

Estimated large deletion: ∼19–21 Mb.

a 10q23.2 (88.2–88.6 Mb).

b 10q23.2 (89.4–89.5 Mb).

c *PTEN* locus (89.6–89.7 Mb).

d 10q23.33 (96.2–96.6 Mb).

e 10q25.1 (108.9–109.3 Mb).

f First event.

g Second event.

## References

[bib1] Ahlers CM, Figg WD (2006) ETS-TMPRSS2 fusion gene products in prostate cancer. Cancer Biol Ther 5: 254–255 16575200 10.4161/cbt.5.3.2603

[bib2] Ashcroft M, Ludwig RL, Woods DB, Copeland TD, Weber HO, MacRae EJ, Vousden KH (2002) Phosphorylation of HDM2 by Akt. Oncogene 21: 1955–1962 11960368 10.1038/sj.onc.1205276

[bib3] Baker SJ (2007) PTEN enters the nuclear age. Cell 128: 25–28 17218252 10.1016/j.cell.2006.12.023

[bib4] Bertram J, Peacock JW, Fazli L, Mui AL, Chung SW, Cox ME, Monia B, Gleave ME, Ong CJ (2006) Loss of PTEN is associated with progression to androgen independence. Prostate 66: 895–902 16496415 10.1002/pros.20411

[bib5] Besson A, Robbins SM, Yong VW (1999) PTEN/MMAC1/TEP1 in signal transduction and tumorigenesis. Eur J Biochem 263: 605–611 10469123 10.1046/j.1432-1327.1999.00542.x

[bib6] Brunet A, Bonni A, Zigmond MJ, Lin MZ, Juo P, Hu LS, Anderson MJ, Arden KC, Blenis J, Greenberg ME (1999) Akt promotes cell survival by phosphorylating and inhibiting a Forkhead transcription factor. Cell 96: 857–868 10102273 10.1016/s0092-8674(00)80595-4

[bib7] Cardone MH, Roy N, Stennicke HR, Salvesen GS, Franke TF, Stanbridge E, Frisch S, Reed JC (1998) Regulation of cell death protease caspase-9 by phosphorylation. Science 282: 1318–1321 9812896 10.1126/science.282.5392.1318

[bib8] Cerveira N, Ribeiro FR, Peixoto A, Costa V, Henrique R, Jeronimo C, Teixeira MR (2006) TMPRSS2-ERG gene fusion causing ERG overexpression precedes chromosome copy number changes in prostate carcinomas and paired HGPIN lesions. Neoplasia 8: 826–832 17032499 10.1593/neo.06427 PMC1715930

[bib9] Datta SR, Dudek H, Tao X, Masters S, Fu H, Gotoh Y, Greenberg ME (1997) Akt phosphorylation of BAD couples survival signals to the cell-intrinsic death machinery. Cell 91: 231–241 9346240 10.1016/s0092-8674(00)80405-5

[bib10] DeMarzo AM, Nelson WG, Isaacs WB, Epstein JI (2003) Pathological and molecular aspects of prostate cancer. Lancet 361: 955–964 12648986 10.1016/S0140-6736(03)12779-1

[bib11] Elo JP, Visakorpi T (2001) Molecular genetics of prostate cancer. Ann Med 33: 130–141 11327116 10.3109/07853890109002068

[bib12] Glinsky GV, Glinskii AB, Stephenson AJ, Hoffman RM, Gerald WL (2004) Gene expression profiling predicts clinical outcome of prostate cancer. J Clin Invest 113: 913–923 15067324 10.1172/JCI20032 PMC362118

[bib13] Goberdhan DC, Wilson C (2003) PTEN: tumour suppressor, multifunctional growth regulator and more. Hum Mol Genet 12(Spec No 2): R239–R248 12928488 10.1093/hmg/ddg288

[bib14] Graff JR, Konicek BW, McNulty AM, Wang Z, Houck K, Allen S, Paul JD, Hbaiu A, Goode RG, Sandusky GE, Vessella RL, Neubauer BL (2000) Increased AKT activity contributes to prostate cancer progression by dramatically accelerating prostate tumor growth and diminishing p27Kip1 expression. J Biol Chem 275: 24500–24505 10827191 10.1074/jbc.M003145200

[bib15] Hughes S, Yoshimoto M, Beheshti B, Houlston RS, Squire JA, Evans A (2006) The use of whole genome amplification to study chromosomal changes in prostate cancer: insights into genome-wide signature of preneoplasia associated with cancer progression. BMC Genomics 7: 65 16573809 10.1186/1471-2164-7-65 PMC1450280

[bib16] Jemal A, Siegel R, Ward E, Murray T, Xu J, Smigal C, Thun MJ (2006) Cancer statistics, 2006. CA Cancer J Clin 56: 106–130 16514137 10.3322/canjclin.56.2.106

[bib17] Kasahara K, Taguchi T, Yamasaki I, Kamada M, Yuri K, Shuin T (2002) Detection of genetic alterations in advanced prostate cancer by comparative genomic hybridization. Cancer Genet Cytogenet 137: 59–63 12377415 10.1016/s0165-4608(02)00552-6

[bib18] Kawai T, Hiroi S, Nakanishi K, Sakurai Y, Torikata C (2004) Abnormalities in chromosome 17 and p53 in lung carcinoma cells detected by fluorescence *in situ* hybridization. Pathol Int 54: 413–419 15144400 10.1111/j.1440-1827.2004.01635.x

[bib19] Koksal IT, Dirice E, Yasar D, Sanlioglu AD, Ciftcioglu A, Gulkesen KH, Ozes NO, Baykara M, Luleci G, Sanlioglu S (2004) The assessment of PTEN tumor suppressor gene in combination with Gleason scoring and serum PSA to evaluate progression of prostate carcinoma. Urol Oncol 22: 307–312 15283888 10.1016/j.urolonc.2004.01.009

[bib20] Korshunov A, Sycheva R, Gorelyshev S, Golanov A (2005) Clinical utility of fluorescence *in situ* hybridization (FISH) in nonbrainstem glioblastomas of childhood. Mod Pathol 18: 1258–1263 15832192 10.1038/modpathol.3800415

[bib21] Kwabi-Addo B, Giri D, Schmidt K, Podsypanina K, Parsons R, Greenberg N, Ittmann M (2001) Haploinsufficiency of the Pten tumor suppressor gene promotes prostate cancer progression. Proc Natl Acad Sci USA 98: 11563–11568 11553783 10.1073/pnas.201167798 PMC58769

[bib22] Liu W, Chang B, Sauvageot J, Dimitrov L, Gielzak M, Li T, Yan G, Sun J, Sun J, Adams TS, Turner AR, Kim JW, Meyers DA, Zheng SL, Isaacs WB, Xu J (2006) Comprehensive assessment of DNA copy number alterations in human prostate cancers using Affymetrix 100K SNP mapping array. Genes Chromosomes Cancer 45: 1018–1032 16897747 10.1002/gcc.20369

[bib23] Majumder PK, Febbo PG, Bikoff R, Berger R, Xue Q, McMahon LM, Manola J, Brugarolas J, McDonnell TJ, Golub TR, Loda M, Lane HA, Sellers WR (2004) mTOR inhibition reverses Akt-dependent prostate intraepithelial neoplasia through regulation of apoptotic and HIF-1-dependent pathways. Nat Med 10: 594–601 15156201 10.1038/nm1052

[bib24] McMenamin ME, Soung P, Perera S, Kaplan I, Loda M, Sellers WR (1999) Loss of PTEN expression in paraffin-embedded primary prostate cancer correlates with high Gleason score and advanced stage. Cancer Res 59: 4291–4296 10485474

[bib25] Merscher S, Marondel I, Pedeutour F, Gaudray P, Kucherlapati R, Turc-Carel C (1997) Identification of new translocation breakpoints at 12q13 in lipomas. Genomics 46: 70–77 9403060 10.1006/geno.1997.4993

[bib26] Mezzelani A, Alasio L, Bartoli C, Bonora MG, Pierotti MA, Rilke F, Pilotti S (1999) c-erbB2/neu gene and chromosome 17 analysis in breast cancer by FISH on archival cytological fine-needle aspirates. Br J Cancer 80: 519–525 10408862 10.1038/sj.bjc.6690387 PMC2362342

[bib27] Miller GJ, Brawer MK, Sakr WA, Thrasher JB, Townsend R (2001) Prostate cancer: serum and tissue markers. Rev Urol 3(Suppl 2): S11–S19 16985995 PMC1476078

[bib28] Ohigashi T, Mizuno R, Nakashima J, Marumo K, Murai M (2005) Inhibition of Wnt signaling downregulates Akt activity and induces chemosensitivity in PTEN-mutated prostate cancer cells. Prostate 62: 61–68 15389810 10.1002/pros.20117

[bib29] Perner S, Demichelis F, Beroukhim R, Schmidt FH, Mosquera JM, Setlur S, Tchinda J, Tomlins SA, Hofer MD, Pienta KG, Kuefer R, Vessella R, Sun XW, Meyerson M, Lee C, Sellers WR, Chinnaiyan AM, Rubin MA (2006) TMPRSS2:ERG fusion-associated deletions provide insight into the heterogeneity of prostate cancer. Cancer Res 66: 8337–8341 16951139 10.1158/0008-5472.CAN-06-1482

[bib30] Qian J, Jenkins RB, Bostwick DG (1998) Determination of gene and chromosome dosage in prostatic intraepithelial neoplasia and carcinoma. Anal Quant Cytol Histol 20: 373–380 9801755

[bib31] Ribeiro FR, Diep CB, Jeronimo C, Henrique R, Lopes C, Eknaes M, Lingjaerde OC, Lothe RA, Teixeira MR (2006a) Statistical dissection of genetic pathways involved in prostate carcinogenesis. Genes Chromosomes Cancer 45: 154–163 16235241 10.1002/gcc.20279

[bib32] Ribeiro FR, Henrique R, Hektoen M, Berg M, Jeronimo C, Teixeira MR, Lothe RA (2006b) Comparison of chromosomal and array-based comparative genomic hybridization for the detection of genomic imbalances in primary prostate carcinomas. Mol Cancer 5: 33 16952311 10.1186/1476-4598-5-33 PMC1570364

[bib33] Schmitz M, Grignard G, Margue C, Dippel W, Capesius C, Mossong J, Nathan M, Giacchi S, Scheiden R, Kieffer N (2007) Complete loss of PTEN expression as a possible early prognostic marker for prostate cancer metastasis. Int J Cancer 120: 1284–1292 17163422 10.1002/ijc.22359

[bib34] Sirvent N, Forus A, Lescaut W, Burel F, Benzaken S, Chazal M, Bourgeon A, Vermeesch JR, Myklebost O, Turc-Carel C, Ayraud N, Coindre JM, Pedeutour F (2000) Characterization of centromere alterations in liposarcomas. Genes Chromosomes Cancer 29: 117–129 10959091 10.1002/1098-2264(2000)9999:9999<::aid-gcc1014>3.0.co;2-q

[bib35] Soller MJ, Isaksson M, Elfving P, Soller W, Lundgren R, Panagopoulos I (2006) Confirmation of the high frequency of the TMPRSS2/ERG fusion gene in prostate cancer. Genes Chromosomes Cancer 45: 717–719 16575875 10.1002/gcc.20329

[bib36] Tomlins SA, Mehra R, Rhodes DR, Smith LR, Roulston D, Helgeson BE, Cao X, Wei JT, Rubin MA, Shah RB, Chinnaiyan AM (2006) TMPRSS2:ETV4 gene fusions define a third molecular subtype of prostate cancer. Cancer Res 66: 3396–3400 16585160 10.1158/0008-5472.CAN-06-0168

[bib37] Tomlins SA, Rhodes DR, Perner S, Dhanasekaran SM, Mehra R, Sun XW, Varambally S, Cao X, Tchinda J, Kuefer R, Lee C, Montie JE, Shah RB, Pienta KJ, Rubin MA, Chinnaiyan AM (2005) Recurrent fusion of TMPRSS2 and ETS transcription factor genes in prostate cancer. Science 310: 644–648 16254181 10.1126/science.1117679

[bib38] van Dekken H, Paris PL, Albertson DG, Alers JC, Andaya A, Kowbel D, van der Kwast TH, Pinkel D, Schroder FH, Vissers KJ, Wildhagen MF, Collins C (2004) Evaluation of genetic patterns in different tumor areas of intermediate-grade prostatic adenocarcinomas by high-resolution genomic array analysis. Genes Chromosomes Cancer 39: 249–256 14732926 10.1002/gcc.20001

[bib39] Ventura RA, Martin-Subero JI, Jones M, McParland J, Gesk S, Mason DY, Siebert R (2006) FISH analysis for the detection of lymphoma-associated chromosomal abnormalities in routine paraffin-embedded tissue. J Mol Diagn 8: 141–151 16645199 10.2353/jmoldx.2006.050083 PMC1867591

[bib40] Verhagen PC, van Duijn PW, Hermans KG, Looijenga LH, van Gurp RJ, Stoop H, van der Kwast TH, Trapman J (2006) The PTEN gene in locally progressive prostate cancer is preferentially inactivated by bi-allelic gene deletion. J Pathol 208: 699–707 16402365 10.1002/path.1929

[bib41] Verhagen PC, Zhu XL, Rohr LR, Cannon-Albright LA, Tavtigian SV, Skolnick MH, Brothman AR (2000) Microdissection, DOP-PCR, and comparative genomic hybridization of paraffin-embedded familial prostate cancers. Cancer Genet Cytogenet 122: 43–48 11104032 10.1016/s0165-4608(00)00276-4

[bib42] Visakorpi T, Hyytinen E, Koivisto P, Tanner M, Keinanen R, Palmberg C, Palotie A, Tammela T, Isola J, Kallioniemi OP (1995) *In vivo* amplification of the androgen receptor gene and progression of human prostate cancer. Nat Genet 9: 401–406 7795646 10.1038/ng0495-401

[bib43] Wang J, Cai Y, Ren C, Ittmann M (2006) Expression of VARIANT TMPRSS2/ERG fusion messenger rnas is associated with aggressive prostate cancer. Cancer Res 66: 8347–8351 16951141 10.1158/0008-5472.CAN-06-1966

[bib44] Whang YE, Wu X, Suzuki H, Reiter RE, Tran C, Vessella RL, Said JW, Isaacs WB, Sawyers CL (1998) Inactivation of the tumor suppressor PTEN/MMAC1 in advanced human prostate cancer through loss of expression. Proc Natl Acad Sci USA 95: 5246–5250 9560261 10.1073/pnas.95.9.5246 PMC20246

[bib45] Wolf M, Mousses S, Hautaniemi S, Karhu R, Huusko P, Allinen M, Elkahloun A, Monni O, Chen Y, Kallioniemi A, Kallioniemi OP (2004) High-resolution analysis of gene copy number alterations in human prostate cancer using CGH on cDNA microarrays: impact of copy number on gene expression. Neoplasia 6: 240–247 15153336 10.1593/neo.3439 PMC1502104

[bib46] Yoshimoto M, Cutz JC, Nuin PA, Joshua AM, Bayani J, Evans AJ, Zielenska M, Squire JA (2006a) Interphase FISH analysis of PTEN in histologic sections shows genomic deletions in 68% of primary prostate cancer and 23% of high-grade prostatic intra-epithelial neoplasias. Cancer Genet Cytogenet 169: 128–137 16938570 10.1016/j.cancergencyto.2006.04.003

[bib47] Yoshimoto M, Joshua AM, Chilton-Macneill S, Bayani J, Selvarajah S, Evans AJ, Zielenska M, Squire JA (2006b) Three-color FISH analysis of TMPRSS2/ERG fusions in prostate cancer indicates that genomic microdeletion of chromosome 21 is associated with rearrangement. Neoplasia 8: 465–469 16820092 10.1593/neo.06283 PMC1601467

[bib48] Zitzelsberger H, Engert D, Walch A, Kulka U, Aubele M, Hofler H, Bauchinger M, Werner M (2001) Chromosomal changes during development and progression of prostate adenocarcinomas. Br J Cancer 84: 202–208 11161378 10.1054/bjoc.2000.1533 PMC2363712

