# Supplementary material for: FISH analysis of 107 prostate cancers shows that PTEN genomic deletion is associated with poor clinical outcome
Source: Br J Cancer. 2007 Aug 14;97(5):678–85. doi: 10.1038/sj.bjc.6603924 (PMC2360375; doi:10.1038/sj.bjc.6603924)
Supplement: Supplementary Table [file 6603924x1.pdf]

Yoshimoto et al. Supplementary Table

Table. Comprehensive FISH raw data from 107 prostatic adenocarcinomas

**Case**	**Number of cells showing *PTEN* gene locus**			**Total of cells**
	**Undeleted**	**Hemizygous deletion**	**Homozygous deletion**	
3	58	35	7	100
6	46	4	0	50
7	90	9	1	100
8	37	10	3	50
10	91	9	0	100
14	88	12	0	100
15	29	33	38	100
16	89	11	0	100
17	34	12	4	50
18	85	15	0	100
19	66	34	0	100
21	36	31	33	100
23	88	12	0	100
27	43	44	13	100
28	48	21	31	100
29	73	27	0	100
30	89	11	0	100
32	64	33	3	100
33	47	3	0	50
34	46	4	0	50
35	29	21	0	50
36	62	34	4	100
37	85	15	0	100
38	57	43	0	100
39	73	24	3	100
40	86	14	0	100
41	49	38	13	100
42	61	26	13	100
44	87	13	0	100
45	64	33	3	100
46	50	50	0	100
49	65	35	0	100
51	88	12	0	100
52	86	14	0	100
53	88	12	0	100
55	94	5	1	100
60	91	9	0	100

continued

Case	Number of cells showing *PTEN* gene locus			Total of cells
	Undeleted	Hemizygous deletion	Homozygous deletion	
61	93	7	0	100
62	71	29	0	100
63	92	8	0	100
68	91	9	0	100
70	87	13	0	100
71	33	67	0	100
72	29	30	41	100
73	55	45	0	100
74	88	9	3	100
76	91	8	1	100
77	38	61	1	100
82	88	12	0	100
83	86	14	0	100
84	90	9	1	100
85	91	8	1	100
86	43	57	0	100
87	37	55	8	100
88	30	39	31	100
91	54	46	0	100
94	66	30	4	100
96	89	11	0	100
99	78	22	0	100
100	46	54	0	100
101	87	12	1	100
102	50	50	0	100
103	94	6	0	100
110	87	13	0	100
111	90	10	0	100
113	44	50	6	100
115	87	13	0	100
116	89	11	0	100
117	50	50	0	100
118	87	13	0	100
119	67	33	0	100
120	89	11	0	100
122	91	9	0	100
123	89	11	0	100
131	95	5	0	100
133	64	31	5	100
135	93	7	0	100

continued

Case	Number of cells showing *PTEN* gene locus			Total of cells
	Undeleted	Hemizygous deletion	Homozygous deletion	
136	38	62	0	100
137	49	3	0	52
138	96	4	0	100
139	91	9	0	100
147	87	13	0	100
149	50	50	0	100
150	36	64	0	100
152	92	8	0	100
153	93	7	0	100
154	88	12	0	100
160	66	34	0	100
164	84	16	0	100
165	39	50	11	100
167	90	10	0	100
170	63	37	0	100
172	93	7	0	100
177	74	26	0	100
178	91	9	0	100
180	62	38	0	100
181	25	75	0	100
182	85	15	0	100
184	37	63	0	100
188	45	5	0	50
190	87	13	0	100
192	93	7	0	100
194	87	13	0	100
195	47	3	0	50
197	91	9	0	100
198	45	5	0	50
199	52	36	0	88
